# Basic Methods for Preparation of Liposomes and Studying Their Interactions with Different Compounds, with the Emphasis on Polyphenols

**DOI:** 10.3390/ijms22126547

**Published:** 2021-06-18

**Authors:** Luka Šturm, Nataša Poklar Ulrih

**Affiliations:** Department of Food Science and Technology, Biotechnical Faculty, University of Ljubljana, Jamnikarjeva 101, 1000 Ljubljana, Slovenia; luka.sturm@bf.uni-lj.si

**Keywords:** model lipid bilayers, bioactive compounds, membrane fluidity, membrane kinetics, essential methods for liposome/vesicle preparation

## Abstract

Studying the interactions between lipid membranes and various bioactive molecules (e.g., polyphenols) is important for determining the effects they can have on the functionality of lipid bilayers. This knowledge allows us to use the chosen compounds as potential inhibitors of bacterial and cancer cells, for elimination of viruses, or simply for keeping our healthy cells in good condition. As studying those effect can be exceedingly difficult on living cells, model lipid membranes, such as liposomes, can be used instead. Liposomal bilayer systems represent the most basic platform for studying those interactions, as they are simple, quite easy to prepare and relatively stable. They are especially useful for investigating the effects of bioactive compounds on the structure and kinetics of simple lipid membranes. In this review, we have described the most basic methods available for preparation of liposomes, as well as the essential techniques for studying the effects of bioactive compounds on those liposomes. Additionally, we have provided details for an easy laboratory implementation of some of the described methods, which should prove useful especially to those relatively new on this research field.

## 1. Introduction

Liposomes are small spherical particles composed mainly from different kind of lipids, which are organised in the form of one or more lipid bilayers. This organisation allows them to be used as a simple approximation to living cells. As the liposomes are fairly simple to produce, relatively stable and also much less delicate to handle than the human cell lines, for example, they can be a very useful tool for determining the basic interactions of bioactive compounds or extracts with the lipid bilayers. Therefore, many medicinal and food-based studies are performed on them [1].

In food industry, one of the most widely used bioactive compounds are plant polyphenols, since they are numerous and quite easy to obtain, even with as simple procedure as ethanol extraction. They are one of the most widely distributed and complex groups of compounds in the plant kingdom, with >8000 phenolic structures currently known. Many of these have strong biological activities, mainly strong antioxidant capacity (free radical scavenging and metal chelation activity). While some polyphenols also showed possible beneficial implications in medicine, e.g., in treatment and prevention of cancer, cardiovascular disease and other pathologies, other showed considerable antimicrobial activity. The most important representatives of such phenols are phenolic acids, hydroxycinnamic acids and flavonoids, while certain individual members of other groups of polyphenols are important as well. Polyphenols are an integral part of the human diet, and they have gained more and more attention because of their potential beneficial effects on human health [2]. However, to understand their full potential, extensive investigations into their interactions with lipid membranes, among other things, have to be carried out.

As the polyphenols have a very diverse and complex structures [2], each molecule has to be studied individually in order to determine its solubility and positioning on or within the lipid membrane [3,4], its permeability through the bilayer [5], and its interactions with the membranes under different conditions [6]. Sometimes, synergistic effects among specific polyphenols and/or other compounds are also studied, in order to better understand the interactions of complex extracts, such as propolis, with the membranes [7]. Since liposomes represent simplified versions of the cells, they are a very suitable solution for studying the effects polyphenols might have on the lipid bilayers.

Indeed, many methods are applied to study how different compounds interact with lipid bilayers, and in this review we focus on the most basic ones: differential scanning calorimetry (DSC), isothermal titration calorimetry (ITC), electron paramagnetic resonance (EPR) spectrometry, nuclear magnetic resonance (NMR) spectrometry, fluorescence anisotropy/polarisation spectroscopy, thiobarbituric acid reactive substance (TBARS) assays, calcein release assays, 4,4-difluoro-5-(4-phenyl-1,3-butadienyl)-4-bora-3a,4a-diaza-s-indacene, boron dipyrromethene (BODIPY 581/591 C11) assays, confocal microscopy, and different methods for determining sizes and surface charges of liposomes. This guide summarizes the theory and practice of the given methods, as well as gives the details for the implementation of some of the mentioned ones.

## 2. Preparation of Liposomes

In order to study the membrane-polyphenol interactions, the lipid membranes have to be obtained/prepared first. The options are many [8]. More complex methods include using the membranes of the human cancer cell lines [9] or obtaining them from living cells, such as erythrocyte ghosts [10,11]. Others include creating them as tethered [12], planar [13] or other none-spherical lipid bilayers [14], while the simplest methods are based on preparing them as simple liposomes [15]. The first artificially made liposomes were created in the 40’s by J.Y. Johnson, whom patented the method for the use in pharmaceutical industry (I. G. Farbenindustrie Aktiengesellschaft). The so called “depots” were obtained when different fats or fatty oils were mixed with various water solutions. A few decades later in the 60’s, unaware of this patent, similar method for creating liposomes were discovered by different researchers, and with the extensive work by Alec Douglas Bangham, one of the first to discovered how to obtained them and also how to use them as a membrane model systems, liposomes quickly gained in popularity [16]. They were soon recognised as robust, simply-created and efficient replacements for model membrane systems and are in use ever since. As the preparation of liposomes is one of the easiest, most cost-affordable options, many studies are based on them.

**Figure 1:**
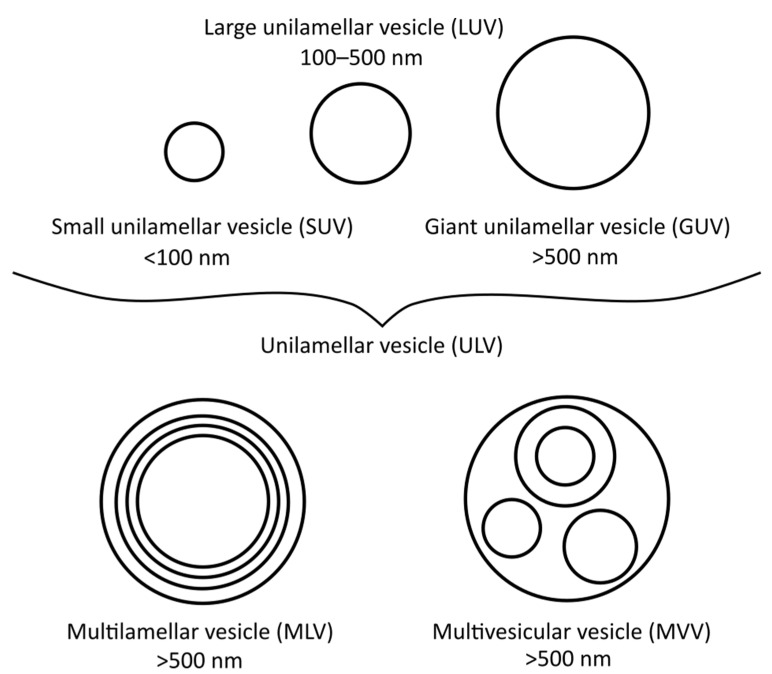
Different vesicle/liposome types and their diameters.

There are many different types of liposomes, some of which are more suitable for specific research techniques than others. The most basic types are small unilamellar vesicles (SUVs), large unilamellar vesicles (LUVs), giant unilamellar vesicles (GUVs), multilamellar vesicles (MLVs), and multivesicular vesicles (MVVs) [17], as illustrated in Figure 1. SUVs, LUVs and MLVs are used the most, especially for DSC (i.e., MLVs), polarisation and anisotropy or for EPR spectrometry measurements (i.e., LUVs and SUVs) [18]. To obtain MLVs, the thin-film or proliposomal methods are usually applied, although other methods can be used as well.

### 2.1. Thin-Film Method

The thin-film method is one of the most widely used liposome preparation techniques. It is based on the creation of a thin film of lipids, which is formed on the inner wall of the rotary evaporator flask. The film thus obtained is latter hydrated with a water or buffer solution. Before the hydration, it is very important that the lipid film, as well as the water/buffer solution, are preheated above the lipids transitional temperature (*T_m_*), when necessary, in order to enable a smoother creation of the bilayer. This, coupled with the vigorous shaking and potential sonication in ultrasonic bath, enables the film to peel of the flask and form liposomes. The liposomes prepared in this way are MLVs of different sizes [19]. The encapsulating substance can be added with the lipids before the formation of the thin film (in the case of lipophilic compounds) or with the water/buffer solution (in the case of hydrophilic compounds). The biggest plus of this method is its high reproducibility even when working with small quantities of compounds, while its biggest minus is its low encapsulation efficiency. It is useful especially for encapsulation of lipophilic components in small, pharmaceutical quantities.

The method has been used by many different researchers [18,20,21,22,23], and the one described here was adapted from Lasch, Weissig [24] and Lasic [25]:

At first, 2–20 mg of lipids (e.g., 1,2-dipalmitoyl-sn-glycero-3-phosphocholine [DPPC], or 1-palmitoyl-2-oleoyl-sn-glycero-3-phosphocholine [POPC]) are weighted in a pre-weighted 10 mL rotary flask, then dissolved in 2–4 mL methanol:chloroform mixture (3:7, *v*/*v*). Solvent is then evaporated at 200–300 mbar (rotary vacuum evaporator) during heating in a water bath at 35–45 °C at appropriate rotation speed. The thin film is formed. It is further dried at high vacuum (5–10 mbar) until constant weight (4 h or overnight (small volumes (<1 mL) can be dried by purging with dry nitrogen)). Before the formation of the liposomes (hydration), the thin film and water/buffer are preheated above *T_m_* of the chosen lipids. This is especially important in the case of lipids with high *T_m_* (e.g., DPPC), whereas in the case of lipids with *T_m_* around room temperature or lower (e.g., POPC) this is not necessary. The water/buffer is then added to yield concentrations between 0.5–10 mg/mL of liposomes. The lipids are hydrated at temperatures above *T_m_* in closed rotary flask (45 min; occasional shaking and sonication in the ultrasonic bath (≈30 s/sonication); addition of small round glass beads if necessary). Finally, the formed MLVs are sonicated in ultrasonic bath for 3 min, stored in plastic microcentrifuge tubes, aerated with nitrogen gas, frozen in liquid nitrogen and stored at −80 °C.

Liposomes stored in this manner are stable for a long period of time. For the preparation of the liposomes with added compounds (e.g., polyphenols), the compounds can be added to the lipids before the preparation of the thin film (i.e., dissolved in the chloroform/methanol mixture, for lipophilic compounds), or before the formation of the liposomes (i.e., dissolved in the hydration solution, for hydrophilic compounds).

**Figure 2:**
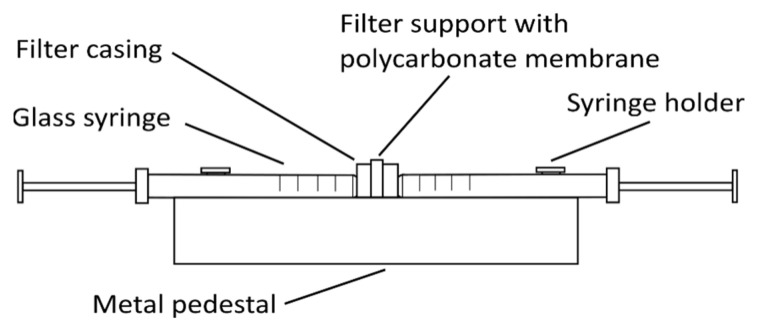
Simple glass syringe hand extruder. The polycarbonate membrane filter of the selected pore size is inserted into the filter support frame. One syringe is then filled with the liposome solution, which is extruded multiple-times from one syringe to the other through the membrane filter, to create small or large unilamellar vesicles.

Other types of liposomes (e.g., SUVs and LUVs) can be prepared from these MLVs by either sonication with a high-intensity ultrasonic cell disruptor (for SUV preparation) or using the extrusion method (for SUV and LUV preparation). For sonication, MLVs are kept in an ice-cold bath during the sonication (e.g., 15 min, 10 s on/off intervals, 40% amplitude, 750 W, for 1 mL MLVs at 1 mg/mL). The SUVs obtained are centrifuged at 15,800× *g* (RCF) for 6 min and then used in the analysis [18]. For the extrusion method (Figure 2), the MLVs need to be extruded multiple times through a thin polycarbonate membrane filter of the required pore size using an extruder, thus creating different sizes of liposomes depending on the filter pore size [21].

### 2.2. Proliposome Method

The proliposomal method might be the simplest method for obtaining the liposomes. In contrast to thin-film method, its biggest minus is its relatively poor reproducibility when preparing smaller quantities of liposomes, while it yields much higher encapsulation efficiencies. The method is based on dissolving the lipids in water and ethanol, while stirring at 60 °C for roughly 10 min, to create a smooth lipid paste. After that, the lipids are cooled down and water/buffer is added in drops, while stirring. The suspension is then hydrated for 1 h as MLVs are formed. The method is very useful for preparation of larger quantities of liposomes, especially for assays like TBARS.

The method was used by different researchers [26,27,28], and the one described here was adapted from Perrett, Golding [29]. In brief:

Between 0.1–1 g of lipids are weighted in a small glass beaker, then 96% ethanol and water/buffer (final lipid:ethanol:buffer ratio of 1:1:2 (*w*/*w*/*w*)) are added. The mixture is heated at 60 °C in a water bath for ≈10 min with stirring on a magnetic stirrer at 600–800 rpm until a fine paste is obtained. The lipid paste is cooled to room temperature and an appropriate amount of water/buffer at room temperature is added in small drops during stirring. The MLVs are formed. Those MLVs are further hydrated during stirring for 1 h at room temperature, following by sonication of liposomes for 3 min in sonication bath. The obtained liposomes are stored for up to 2 days in the fridge (4 °C), or are transferred to centrifuge tubes, aerated with nitrogen gas, frozen in liquid nitrogen and stored at −80 °C.

To obtain proliposomes with encapsulated compounds, the compounds can be added with the ethanol before (for lipophilic compounds), or with the water/buffer during the formation of the liposomes (for hydrophilic compounds).

### 2.3. Injection Methods

There are many variations to this method, although the injection of the lipid suspension (for either hydrophobic or hydrophilic organic solvents) into the water phase is characteristic of all of them. In general, the main advantages here are the simplicity of the preparation procedures, and the preparation of large quantities of liposomes, although this also requires large amounts of usually expensive compounds [25,30]. In this review we will briefly discus the ethanol and ether injection methods.

#### 2.3.1. Ethanol Injection

Ethanol injection method is used for preparing liposomes ranging between 30–170 nm, and is usually used to prepare SUVs. The size itself depends on the concentration of lipids and the injection speed. When preparing liposomes using this method, the lipids dissolved in an organic solvent (in this case ethanol) are injected into the water phase during stirring, and then the solvent is removed. The solution is then left hydrating during stirring for another 15 min. The ethanol can be removed from the liposome suspension either by rotary evaporation or by centrifugation through a silica gel column.

The limitations of this method are very poor encapsulation efficiency of hydrophilic compounds, the relatively limited solubility of lipids in ethanol, and the limited concentrations of lipids in the final solution due to the high ethanol content in it. Also, ethanol concentration should not exceed 7.5%, in order to prevent liposome destabilisation, which impacts the amount of the lipids that can be added [25,31,32]. The method is otherwise quite useful for preparation of large quantities of liposomes on industrial scale [33].

#### 2.3.2. Ether Injection

Ether injection method is very similar to the ethanol injection method, with the important exception, that the lipid solvent used in this case, ether, does not mix with water at all. This, coupled with higher lipids solubility in ether opposed to ethanol and the fact that ether does not disrupt liposome formation, enables the preparation of higher concentrations of liposomes. After injection, ether is removed from the solution in the same manner as ethanol.

There are also some downsides to this method, as ether and water phases need to be at different temperatures during the injection procedure, ether might affect the encapsulation of some compounds, and the liposomes formed have very heterogeneous shapes. Injecting the lipid suspension into the water/buffer should also be slower than for ethanol injection method, and it is advised to do this under vacuum. However, the liposomes produced using this method have higher encapsulation efficiencies. In contrast to ethanol injection method, LUVs are formed rather than SUVs [25,30]. A fine example of this method was applied by [34].

### 2.4. Emulsification Method

The emulsification method is similar to the injection methods, as the lipids are again dissolved in organic solvent and then both organic and water phases are mixed together. In contrast to the injection method, however, smaller quantities of water phase are added to the organic phase (i.e., at an inverted organic/water phase ratio), and not the other way around. Also, the organic phase is not removed soon afterwards—instead, an emulsion of water in organic phase is formed. The lipids form a monolayer around water droplets, and then the organic solvent is evaporated. With the evaporation of the organic solvent, liposomes are formed around water particles. The mayor plus of emulsification method is, that it provides higher encapsulation efficiencies compared to the injection methods. Among many different versions of emulsification method known, the reverse-phase evaporation is one of the most basic ones [25,30].

The reverse-phase evaporation method was used by different researchers [35,36], and the one described here was applied by Cortesi, Esposito [37]. Briefly:

First, 40 mg of phosphatidylcholine is dissolved in 1 mL chloroform/methanol (2:1, *v*/*v*) in a 25-mL round-bottomed flask, followed by evaporation of solvent by rotary evaporation. Then either a different (i.e., ethanol or diethyl ether) or the same organic solvent is added, to yield a final volume of 8 mL. Further, additional 2 mL of a water/buffer solution is added and organic phase is evaporated by rotary evaporation at 40 °C under reduced pressure. The acquired aqueous suspension is sonicated in ultrasonic bath, during additional shaking. The liposomes obtained in this way are mainly LUVs, with a mean diameter of around 320–480 nm. To prepare smaller liposomes, these liposomes can be sonicated or extruded, as indicated before. For encapsulation of compounds, lipophilic molecules are dissolved in the organic phase together with the lipids, while the hydrophilic molecules are added together with the water phase.

## 3. Spectroscopic Determination of the Position of the Compounds in the Bilayers

There are many spectroscopic methods to study the membrane–polyphenol interactions and polyphenol positioning in the lipid bilayer, yet some are more useful than others. Among these methods, polarisation and anisotropy measurements, as well as the EPR spectrometry, are the two methods most widely applied. They are useful especially for determining the position of the chosen compound in the lipid bilayer and how this affects the membrane fluidity.

### 3.1. Polarisation and Anisotropy Measurements

Measurements of the polarisation and anisotropy can be carried out to determine the position of a studied compound in the bilayer and its fluidifying effects on it. This is done by indirectly measuring the rotational speed of a chosen fluorescent compound, which is intercalated (together with the studied compound) in the liposomal membrane [38]. For these measurements, fluorophores 1,6-diphenyl-1,3,5-hexatriene (DPH), which is incorporated between the acyl tails of the bilayer lipids (i.e., deep within the membrane bilayer), and 1-(4-trimethylammoniumphenyl)-6-phenyl-1,3,5-hexatriene ρ-toluenesulfonate (TM-DPH), which is incorporated into the water–lipid interphase between the bilayer lipid polar head groups (i.e., more superficially), are usually used [39], but other fluorophores can be used as well [40]. The spectrophotometer measures light diffraction caused by the rotational speed of DPH or TM-DPH, which depends on the effect studied compound has on the bilayer. In this way it is possible to determine the compounds positioning and its effect on fluidity of the bilayer.

The method described here is adapted from Lakowicz [38], and has been widely applied [23,41,42,43]. In brief:

First, stocks solutions of 1 mM DPH and 2 mM TM-DPH DMSO, as well as 1 mg/mL SUVs via thin-film method as described in Section 2.1, and 10 mM solution of chosen compound in either aqueous solution or ethanol are prepared. SUVs (250 µL; 1 mg/mL) are loaded with 2.5 µL stock solution of either DPH or TM-DPH in a 10-mm-path cuvettes, followed by dilution with aqueous solution to 2.5 mL (final concentration of 1 mg/mL). This is followed by gradual addition 10 mM solution of chosen compound to the cuvettes, while aqueous solution or ethanol are used as negative control. Usually, the intermediate volumes in the cuvette’s are as follow—1, 2, 3, 4, 5, 10, 15, 20, 30, 40, 50, 75, 100 and 125 µL. After the addition, samples are incubated for 10 min and then polarisation and fluorescence emission anisotropy are measured, using a fluorescence spectrophotometer with slits with nominal band-pass of 5 nm for both excitation and emission at an excitation wavelength of 358 nm, with the excitation polariser in the vertical position, while the vertical and horizontal components of the polarised light are recorded using a monochromator at 410 nm. The experiments are usually carried out at two temperatures, one below and one above the *T_m_* of the chosen lipids (e.g., at 25 °C and 47 °C for DPPC SUVs), while in the case of SUVs made from lipids like POPC, which has a *T_m_* around −2 °C, only one temperature is chosen (e.g., 25 °C). The final concentrations in the cuvettes under these loading conditions are 0.1 mg/mL liposomes and 1 µM or 2 µM DPH or TM-DPH, and are slowly lowered with the addition of compound solution. The emission fluorescence of DPH and TM-DPH in aqueous solution is generally negligible, as in our case here. The anisotropy (*r*) is usually used for data processing, and it is calculated using the built-in software of the spectrophotometer, according to Equation (1) [38]:

(1) $$r=I_{HH}-GI_{HV}/\left(I_{HH}+2GI_{HV}\right)$$

While the polarisation (*P*) is calculated according to Equation (2) [38]:

(2) $$P=I_{HV}-I_{HH}/\left(I_{HV}+I_{HH}\right)$$

where *I_HH_* and *I_HV_* are the horizontal and vertical emission intensities, respectively. The *G*-factor is the ratio of the sensitivities of the detection system for the horizontally (*I_HH_*) and vertically (*I_HV_*) polarised light, and it is determined for each sample separately, according to the Equation (3) [38]:

(3) $$G=\frac{I_{HV}}{I_{HH}}$$

The lipid order parameter (*S*) is calculated from the anisotropy, using Equation (4) [44]:

(4) $$S=\frac{{\left[1-2\left(r/r_{0}\right)+5{\left(r/r_{0}\right)}^{2}\right]}^{1/2}-1+r/r_{0}}{2\left(r/r_{0}\right)}$$

where *r*_0_ is the fluorescence anisotropy of DPH in the absence of any rotational motion of the probe. The theoretical value of *r*_0_ for DPH and TM-DPH is 0.4.

### 3.2. Electron Paramagnetic Resonance Spectrometry for Determination of Compound’s Position

Electron paramagnetic resonance (EPR) spectrometry is a technique in which the free/unpaired electrons, which are usually present in ions, free radicals and spin probes, are exposed to a strong magnetic field. Due to the effects of the field, the spin of these free electrons is aligned either parallel or anti-parallel to the magnetic field. When the magnetic field strength is changed, the position and population of energy levels of the electrons changes as well, which leads to a resonance with the microwave radiation of the instrument. This change in electron energy is called the electron resonance and is reflected on the energy change (difference) in the magnetic field. The difference in the magnetic field is influenced by magnetic shielding and coupling of the environment and its dynamics, and is the parameter measured by the machine. The signal received depends on a number of factors, which are mainly the position of the electron in the molecule and the environment in which the molecule is present. With the obtained data, it is possible to determine the positioning and interactions of the chosen compound in different parts of the bilayer membrane. To determine the position and interactions of polyphenols within bilayer membranes, spin labels or spin probes are usually used, as polyphenols and lipids do not usually contain such free or unpaired electrons [45]. EPR could hardly be counted among the simplest of methods, since it requires expensive machinery and specific knowledge for analysis of data, but since it is massively used, it could be counted among basic methods for studying the membranes, including liposomes.

EPR is suitable for investigation of liposomes created from various lipids, e.g., DPPC, POPC or mixture of phosphatidylcholine:sphingomyelin, and it has been applied by different researchers [46,47,48]. The method described here was adapted from Abram [18] and Ulrih, Maričić [43].

In brief: aliquots of 35 µL 0.1 mM solutions of specially designed spin-label methyl esters of doxyl palmitic acid, with the doxyl group on carbon 5 (MeFASL(10,3)) (monitoring properties near water-lipid interface) or carbon 13 (MeFASL(2,11)) (monitoring properties in the middle of the bilayer) of the alkyl chain (counting from the methyl group) in 99% ethanol are dried in glass test-tubes to obtain a thin film on the walls of the tubes. This is followed by addition of 50 µL SUVs prepared via thin-film method as described in Section 2.1 (e.g., 5 mg/mL) to the tubes, with thorough mixing with a vortex mixer (10 min). Then 3.5 µL of the polyphenol suspension (e.g., 15 mM) in 99% ethanol is added and solution is mixed for 5 min. The mixture is transferred to a 1-mm glass capillary tubing for measurement in an EPR spectrometer over the desired temperature range (e.g., 10–50 °C for DPPC, at 5 °C intervals).

The parameters of the spectrometer used for DPPC SUVs were: centre field, 332 mT; scan range, 10 mT; microwave power, 20 mW; microwave frequency, 9.32 GHz; modulation frequency, 100 kHz; and modulation amplitude, 0.1 mT. The final concentration of the polyphenols in this mixture was 1 mM. The empirical correlation time (*τ_emp_*) can be calculated from the EPR spectra. In this manner, rough estimations of the ordering and dynamics of the spin probe motion can be acquired, which reflects the motion of the surrounding lipids, to define the changes caused by the incorporation of the polyphenols into the SUVs. A short *τ_emp_* indicates a dynamic environment inside the membrane based on the fast motion of the nitroxide group of the spin probe, which in this case points to low ordering of the phospholipid acyl chains. This in turn indicates high membrane fluidity. As the EPR spectra data measured directly from the spectra only provide information about the mean membrane fluidity, for more precise analysis of the bilayer membrane characteristics, computer simulation of the EPR spectra line can be performed, using, e.g., the EPRSIM WIZ 6.2.2. Software.

### 3.3. Nuclear Magnetic Resonance Spectrometry for Determination of Compound’s Position, Permeability and Its Effects on the Liposomes

Nuclear magnetic resonance (NMR) is a technique which uses the spin of the atom nuclei, or better said, the changes in magnetic field energy these nuclei generate, to determine the environment and its dynamics of the chosen molecule(s). It is very similar to EPR, but unlike EPR, which functions based on the free/uncoupled electrons (see Section 3.2 above), NMR works on the basis of nuclei with nonzero nuclear moment, in other words their spin, which generates a weak magnetic field. When exposed to the magnetic field created by the machine, the magnetic field of the nuclei is either aligned parallel with the machine’s (lower energy state) or opposite to it (higher energy state). When a certain frequency is applied, these nuclei start to resonate, which means their magnetic fields orientation starts to switch from the lower to higher energy states. This change in the energy of the magnetic field is then measured and results interpreted. Since the environment and its dynamics of the nuclei also determine the difference in energy change of magnetic field, it is possible to estimate what is happening around the observed nuclei. It is important to note, however, that to measure those changes, the observed molecule needs to include at least one atom with a nonzero spin. This means that the atom needs to have either odd number of protons or an odd number of neutrons. Any atom which meats these requirements will suffice, but for the analysis in biochemistry atoms like ^13^C, ^1^H and ^31^P are usually used. Out of two distinctive NMR spectrometers in use, pulsed or Fourier transform NMR is almost exclusively applied (the other one being continuous-wave NMR) [49,50,51].

In biomembrane biochemistry different NMR techniques are applied in order to study different parts/effects of the membrane. It is possible to determine things such as behaviour and motion of the phospholipid groups of the lipids [52], the interactions between lipids and different molecules, like polyphenols [51,53,54], to study the liposome polymorphism, shape and size [55] and even to study the molecular exchange trough liposome membranes [56]. For these studies, many different lipids can be used, but the ones that mimic the living membranes, such as POPC or 1,2-dimyristoyl-*sn*-glycero-3-phosphocholine (DMPC), are used the most [51,53,57]. The specifics and preparation of samples differ from each other depending on the technique we are using, but some basic preparation steps are integral for them all—one of those being the preparation of almost all liposomes in D_2_O water, to exclude the otherwise too strong hydrogen signal from the non-deuterium water. Also, deuterium small quadropole moment makes D_2_O an ideal probe for the membrane lipids [49].

The shortcomings of the NMR methods are relatively few, two of the biggest ones being the price of the machines and the complexity of data analysis. Since many times the results cannot be interpreted from NMR data alone, different techniques, some also mentioned in this article, are used to further confirm the observations and draw firmer conclusions [50].

## 4. Determination of the Effects of Compounds on the Liposomes

### 4.1. Determination of the Permeability of the Liposomal Bilayers: The Calcein Release Method

The permeability of a lipid bilayer is an important property of all membranes, and it usually depends on membrane fluidity. The effect of the chosen compound on the lipid membrane also depends on its permeability through it, and vice versa, while this information is integral to the analysis of drug application. The calcein release method is the most widely used method for such determination [58].

Calcein is a fluorescent dye that is being used, besides for determining the permeability of lipid membranes, for monitoring the interactions between closed lipid membranes and targeted compounds. It has the unique property of self-quenching in high concentrations, which means that it emits fluorescent light only after being released from liposomes. The data predominantly show that the speed of calcein release from liposomes is mostly dependent on the fluidity of the bilayer, with faster release in the case of more fluid membranes [59].

This technique was used by different researchers and can be used for studying liposomes, model membranes or membranes of living cells [59,60,61,62]. The protocol described here was used by Kuboi, Shimanouchi [63] and Maherani, Arab-Tehrany [64], where calcein release from liposomes can be determined using LUVs or SUVs that contain 20–100 mM calcein encapsulated within them. In brief:

MLVs containing calcein are prepared via thin-film method as described in Section 2.1 (calcein is added as an aqueous solution), while unilamellar liposomes are obtained via extrusion through a polycarbonate membrane (to obtain LUVs) or sonication (to obtain SUVs). The untrapped calcein molecules are removed by size-exclusion chromatography, using commercial chromatography columns (e.g., Sepharose-4B columns (10 × 150 mm) or Sephadex-G75 columns (10 × 200 mm)) which were pre-equilibrated with the required buffer (e.g., phosphate buffer). This is followed by elution of liposomes with buffer (e.g., 100 mM Tris-HCL, 150 mM NaCl (pH 7.5)), and later by dilution of liposome suspension with buffer to a final concentration of 100 mM lipid. Then a chosen compound is added to the liposomes containing calcein (e.g., 10 µM of a chosen polyphenol molecule) and several measurements of the fluorescence of the entrapped and released calcein are performed, using a fluorescence spectrometer, with emission and excitation wavelengths set at 490 nm and 520 nm, respectively (at different time intervals). At the end, 3% Triton X-100 is added to the solution (cell lysis and release of all remaining calcein) and a final measurement is taken.

Due to the self-quenching effect of calcein, the fluorescence of the liposomes containing calcein should be low. The amount of calcein released over time (*t*) is calculated according to Equation (5) [63]:

(5) $$RF\left(\%\right)=100(I_{t}-I_{0)}/\left(I_{max}-I_{0}\right)$$

where *RF* is the fraction of calcein released, *I*_0_, *I*_t_ and *I_max_* are the fluorescence intensities measured at the beginning of the experiment, at time *t*, and after addition of 3% Triton X-100, respectively. First order kinetics were employed to analyse calcein release, according to the Equation (6) [63]:

(6) $$RF=RF_{max}\left(1-\left(-e^{(-k_{pert}t}\right)\right)$$

where *RF_max_* and *k_pert_* represent the maximum value of *RF* and the release rate constant, respectively. To determine *RF_max_* all experiments were run until the value of calcein fluorescence reached a constant value. The faster the release kinetics of calcein, the more fluid the membrane.

### 4.2. Spectroscopic Methods for Measuring Antioxidant Effects on the Model Lipid Membranes

Spectroscopic methods also have important roles in determination of the effects of compounds on lipid membranes, such as antioxidant or peroxidation effects on the lipids. One of the most useful and robust methods to determine lipid peroxidation is the so-called thiobarbituric acid reactive substances or TBARS assay. This assay is used to determine peroxidation state of different lipid systems, and can also be used to determine the potential of a compound to inhibit peroxidation of the membrane lipids caused by different environmental factors, e.g., UV radiation [65]. The other more recently introduced methods for determination of the antioxidant and peroxidation potential of compounds towards lipid membranes are the BODIPY 581/591 C11 technique [66] and EPR spectroscopy [46]. Despite being more complicated than TBARS assay, they are still widely used, and can thus be counted among more basic methods for such determinations.

#### 4.2.1. Thiobarbituric Acid Reactive Substances

The TBARS assay is a method where boiling the oxidised lipids (e.g., liposomes) in a mixture of different acids results in the appearance of a pink colour. The colour is generated when the thiobarbituric acid reacts with malondialdehyde, which is formed during the peroxidation—more colour means more oxidation damage lipids. This assay cannot be used to define the complete peroxidation of the liposomes, but it can be used to show the approximate effects of lipid oxidation [67]. TBARS assay is a technique implemented by many researchers [46,68,69] duo to its simplicity. The assay described here is adapted from Pelle, Maes [70], and can be used to determine the antioxidative effect of a chosen compound on a liposomal bilayer. In brief:

The liposomes (both empty and with the chosen compound (e.g., 20:1—lipid:compound molar ratio)) are prepared by thin-film method or proliposomal method, as described in Section 2.1 or Section 2.2, respectively. Then 2 mL liposomes (e.g., 10 mg/mL) are pipetted into a special 2.5-mL UV cuvettes with caps. Exactly ½ of them are positioned (including both, empty and encapsulated liposomes) horizontally with the transparent side facing the UV light (e.g., wavelength 254 nm, at 50 Hz), while the other ½ are stored in the dark (negative control). The cuvettes with the samples are then incubated for 24 h. For each measurement, small aliquots (0.2 mL) are taken out of the cuvettes at a certain time interval (usually 0, 1, 2, 4, 6 and 24 h) and put into a test-tube with a plastic screw cap, then 3 mL 20% trichloroacetic acid and 1 mL mixture of 1% thiobarbituric acid and 10% perchloric acid are added (since thiobarbituric acid is not completely soluble at room temperature, the mixture of thiobarbituric and perchloric acid needs to be preheated and stirred at ~70 °C, before being pipetted into the test-tubes). This is followed by heating the samples mixture in a closed test-tube for 25 min at 100 °C in a water bath, and then the reaction is stopped by placing the test-tubes into an ice bath (1–3 °C) for 5 min. The tubes are then centrifuged at 1015× *g* (RCF) for 8 min, the supernatant is carefully transported into polystyrene cuvettes and the absorbance is measured at 532 nm. For the blank sample, 0.2 mL of the buffer used for the preparation of the liposomes is added, instead of the liposomes.

#### 4.2.2. The BODIPY 581/591 C11 Method

The fluorescent probe BODIPY can be used to monitor trafficking of different compounds through multiple cellular cycles and membrane dynamics, and to determine of many other cellular properties [71]. In the case of liposomal model membranes, the BODIPY fluorophore can be used to determine the peroxidation dynamics inside lipid membranes after addition of the compounds under investigation. Different BODIPY probes have been developed for specific uses, with BODIPY 581/591 C11 developed to determine the peroxidation dynamics of bilayers. The BODIPY 581/591 C11 probe is a fatty-acid analogue that is intercalated into the lipid bilayer and changes its emitted fluorescent light from red to green according to the oxidation of the phospholipid fatty acids by reactive oxygen species [72].

The BODIPY 581/591 C11 technique has been widely used in research [73,74,75], while the method described here is relatively simple to use and requires a fluorimeter to measure the light emitted by the probe. The protocol is adapted from [76]. In brief:

Aliquots of SUVs (empty and with encapsulated compound), prepared via thin-film method, as described in Section 2.1, are loaded with the BODIPY 581/591 C11 probe (from a 2 mM stock solution in DMSO) and diluted to 2.5 mL with buffer (e.g., 10 mM HEPES, pH 7.0) to a final concentration of 1 mg/mL lipids and 1 µM BODIPY 581/591 C11. This is followed by incubation of samples at 25 °C for 15 min and inducing of peroxidation of the SUVs by addition of 20 µL 1 mM CuCl_2_ solution. Finally, the fluorescence emission from the probe at excitation wavelength of 500 nm and emission wavelength of 520 nm is measured.

The inhibition of peroxidation is calculated by comparison of the peroxidation levels in the control sample (with only the lipids; expressed as 100% peroxidation) and the samples with different amounts of the compounds under investigation, expressed as percentages. The assay is based on the sensitivity of the BODIPY 581/591 C11 probe to oxidation by radicals that are formed from lipid hydroperoxides.

#### 4.2.3. Electron Paramagnetic Resonance Spectrometry for Determination of Antioxidant Activity

In the field of membrane research, EPR spectrometry can be used not only to determine the position of a compound within a lipid bilayer, as described in Section 3.2, but also for determining the antioxidant activity a chosen compound has on a lipid membrane. This is achieved by using the so-called EPR spin trapping. With this method, it is possible to capture the short-lived radicals in the liposome suspensions after UV radiation, and to transform them into more stable radicals, which can then be measured by EPR spectroscopy. The method can also define the type of radicals and the kinetics of their formation and transformation [46]. The method is used by different researchers [77,78] and the one described here is adapted from Balanč, Ota [46]. In brief:

Exactly 27 µL of proliposome suspension, prepared as described in Section 2.2 (e.g., 20 mg/mL), 4.5 µL of 1 M solution of the spin trap compound (in this case 5-(diethoxyphosphoryl)-5-methyl-1-pyrroline-N-oxyde—DEPMPO) dissolved in aqueous solution, and 13.5 µL of the compounds under investigation (in ethanol or 10% (*w*/*w*) ethanol solution) are mixed together. The suspension is then irradiated with UV light (wavelength, 254 nm) for 5 min. Finally, the EPR spectrum is measured, as described in Section 3.2 (the parameters of the spectrometer are: centre field, 332 mT; scan range, 15 mT; microwave power, 20 mW; microwave frequency, 9.32 GHz; modulation frequency, 100 kHz; and modulation amplitude, 0.1 mT). The same procedure is also applied to the suspension without the compounds (negative control), and the data are compared. The EPR spectra intensity is proportional to the amounts of the radicals captured. Therefore, the ratio between the EPR spectra intensity in the presence of the compounds under investigation and their absence, provides information about their antioxidant activity on the lipid bilayer. The EPR spectra intensities are obtained by double integration of the EPR spectra, while the nature of the radicals captured is obtained by computer simulation of these spectra. Thus, the hydroxyethyl radical adduct is a measure of OH-radical production. The data obtained here provide information on the nature of the radicals formed by the UV irradiation in the presence of the phospholipids used for liposome formulation and the inhibitory effects of the compounds under investigation, as defined for these particular radicals irrespective of the possible influence of ethanol on liposome integrity [46]. Due to the relatively expensive equipment and complexity of the data processing, this method is not used as frequently as the previous two methods described above.

### 4.3. Surface Charge Measurements

The surface charge of model lipid membranes has an important role in the interaction dynamics, not only in terms of membrane-compound interactions, but also on the membrane-membrane, or in the present case, the liposome-liposome interactions. The surface charge of a lipid membrane mainly depends on its lipid composition and the pH of the environment, and the same goes for any other molecule [79]. For example, at physiological pHs, the polyphenols (their OH-groups) are usually in the de-protonated state, and are thus likely to interact with the outer parts of the lipid membranes only (i.e., water–lipid interface; polar headgroups), while on the other hand, at relatively acidic pHs (e.g., 3.5), the polyphenols are usually protonated, and are thus more likely to penetrate deeper into the lipid bilayer [80,81]. In the case of zwitterionic lipids (e.g., DPPC and POPC), the liposomes formed have almost no apparent charge at physiological pH, while for charged phospholipids (e.g., phosphatidylserines, phosphatidylinositols), the net charge of the liposomes is determined by the sum of their total charge [79,82]. Knowing the charge of both liposomes and the chosen compound is thus very important from the membrane-membrane and membrane-compound interactions point of view. Not only does it dictates what the effect of the compound on the membrane is going to be, or what part of the membrane the compound is going to interact with, but it also determines if the prepared liposomes are susceptible to aggregation. Usually, charged liposomes are more stable in a solution than neutrally charged ones, which will often fuse with each other [83]. Despite the surface charge complex functionality and demanding study, it can be simply measured using a particle size and zeta potential analyser, like Zetasizer. The measurements are simple:

- Pipetting 20–50 µL of liposomal solution to the bottom of a specially designed zeta potential measuring cuvette, filled with filtered ultra-clean water (pore size, 0.45 µm)
- Measuring the zeta-potential (in the case of aqueous liposomal suspensions, water is chosen as the solvent, the cuvette-type settings are selected according to the cuvette in use, and the number of measurements is defined by the machine software)

### 4.4. Size Measurements

The size of all lipid membranes, especially of the liposomes, is an important parameter, as its role on determining the effects of different compounds on the lipid bilayer is not negligible. It especially affects the inter-molecular forces, which also reflects on the fluidity and permeability of lipid bilayers. Indeed, size has a particularly important role in the development of drug-loaded vesicles or vesicles used in functional foods [55]. Size measurements can be obtained by different methods, and two of the simplest and most commonly used ones are presented here: dynamic light scattering and microscopy.

#### 4.4.1. Dynamic Light Scattering

One of the most common methods used to measure average liposome sizes in suspensions is dynamic light scattering. The method applies spectrophotometry to measure photon correlation, thus providing measures of particle size. For this analysis, a particle size and zeta potential analyser can be used, as was in the case of our studies, and those made by different researchers [21,84,85]. Otherwise, measuring the liposome size with this method is relatively simple and fast: approximately 2 mL of aqueous liposome suspension at the chosen concentration (e.g., 0.5–20 mg/mL lipids) is put into a 2.5-mL polystyrene cuvette, or 20–50 µL of the same sample is put into the specially designed zeta potential measuring cuvette, filled with the filtered aqueous solution, exactly as for the zeta potential measurements described in Section 4.3. The parameters are then chosen based on the solution, cuvette type and desired temperature, and the measurements are taken. This method usually yields good data, at least for samples with homogenous particle sizes. For samples with very heterogeneous particle sizes, on the other hand, high polydispersity indices are obtained. This can be problematic, however, since due to the mathematical plotting of the average liposome sizes, the results can differ from the actual ones. This technique can also be problematic for the liposomes larger than 10 µm, since some machines have problems quantifying such particles. Thus, a reference method should be used for comparison in order to prove the correct measuring.

#### 4.4.2. Light Microscopy

**Figure 3:**
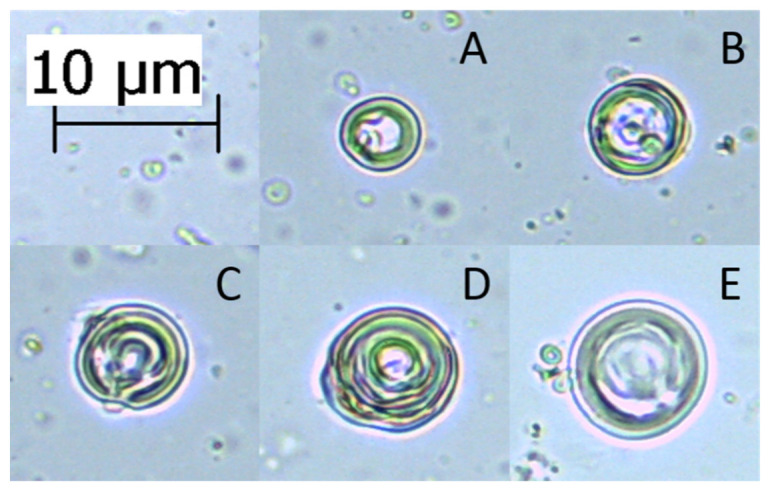
Light microscopy images of different types and sizes of dipalmitoyl-phosphatidylcholine (DPPC) liposomes and its mixture including chrysin, quercetin, caffeic acid and *trans*-ferulic acid (DPPC:chrysin/caffeic acid/*trans*-ferulic acid [n/n], 5:1; DPPC:quercetin [n:n], 10:1) as suspensions in HEPES buffer (20 mM, pH 7.0) (magnification, 400×). (**A**,**C**–**E**) Multilamellar vesicles. (**B**) Multivesicular vesicle. All images include, besides the one bigger liposome in the centre, a larger number of smaller liposomes for size comparison.

As dynamic light scattering can sometimes produce less accurate data, as described in Section 4.4.1, microscopy measurements can be performed for comparison. This technique allows individual liposomes of non-standard sizes to be appropriately measured, and thus provide improved determination of the maximum liposome sizes, especially those above 10 µm. The drawback of this method, however, is that it is only acceptable for MLVs and MVVs, since those liposomes are the only ones which can be detected under a light microscope. Some larger liposomes, such as GUVs, might be measured as well, but need to be phase contrasted with specific dyes or dark-field microscopy must be used, while LUVs and SUVs cannot be measured at all. Despite optical microscopy not being the most popular for visualisation of liposomes among scientists, it is still sometimes used [86,87]. For light microscopy the measurements are relatively simple: a small drop of the liposome suspension (e.g., 10 µL) at a suitable concentration is pipetted on a microscope slide and the drop is covered with a thin coverslip. It can then be examined under the light microscope with a camera attachment, under 400× or 1000× magnification. Chosen liposomes in the sample images are then measured (Figure 3). Despite the simplicity of this method, measuring liposome sizes in this way can introduce errors of bias, based on the choosing of the liposomes to measure. Thus, the method is best used only for determining the sizes of the largest liposomes produced.

#### 4.4.3. Transmission Electron Microscopy

**Figure 4:**
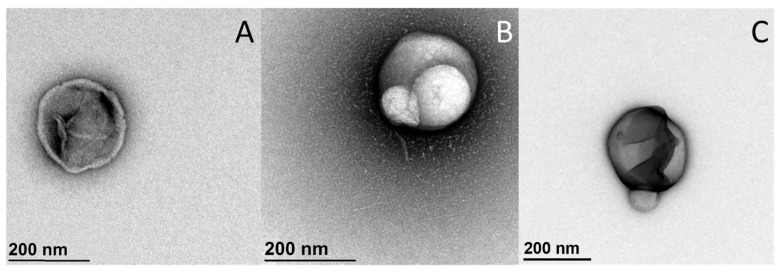
Representative transmission electron microscopy images of dipalmitoyl-phosphatidylcholine (DPPC) liposomes without (**A**) or with (**B**,**C**) addition of epigallocatechin-3-gallate (DPPC:compound [n/n], 1:1). The liposome in (**C**) was previously used for differential scanning calorimetry measurements (i.e., heated twice to 70 °C), while those in (**A**,**B**) were not.

Transmission electron microscopy (TEM) is one of the most widely used methods to display the size and shape of the liposomes. It allows the changes of liposomes to be monitored after addition of the compounds under investigation (Figure 4), or their sizes to be measured. Yet since the transmission electron microscope might be one of the most expensive devices in the laboratory, only few studies can afford this on a daily basis. Also, because of its complexity, it requires a highly trained team (often set-up as a ‘Facility’ within a Department) in order to operate it efficiently. As the procedures of preparation of liposomes vary greatly, it would not be practical to review them here, thus only few are mentioned. The most commonly used TEM technique, which is also used in our laboratory, is negative-staining TEM, while techniques such as cryo-TEM and freeze-fracture TEM can also be used. Negative-staining TEM is the simplest and cheapest of these techniques, as it requires the simplest of the TEM technology (although it still remains particularly complex) [88]. This method is useful for determining sizes of individual liposomes, but less useful for determining their average size in the suspension, as many photographs need to be taken in order to have good enough representativeness. It might better be used in the same way as the light microscopy technique.

### 4.5. Calorimetric Methods for Determination of the Phase Transition of the Membranes

#### 4.5.1. Thermodynamic Profile of Thermotropic Phase Transition Using Differential Scanning Calorimetry

Differential scanning calorimetry (DSC) is a technique that can be applied to the study of the thermotropic phase transition of liposomal bilayers, among others. The studies are based on the generation or consummation of heat, which occurs when the lipids in the membrane reorganise from gel to liquid state. Many membrane studies have focussed on the transition temperatures of lipids only, which are relatively easy to determine, while others have focused on compound-lipid bilayer interactions, where the change in the lipid bilayer organisation due to interactions with the particular compounds can be studied [18,89,90]. Based on the data obtained, the kinds of interactions that take place between the compounds and the lipid membranes can be inferred. DSC is widely applied due to its relative simplicity, good reproducibility, and rapid production of large amounts of data [91,92].

To follow the changes in the gel–to–liquid phase transition of liposomes, under the influence of added substances, different ratios of different compounds or their combinations are used, as different lipid:compound molar ratios yield different data. DSC can be carried out on a range of various DSC systems, which include DSC models with volumetric cells (two cells filled with aqueous samples, using a long syringe) and DSC models with furnaces (sample and reference contained in an aluminium pan) [92,93]. In the case of Ota, Abramovič [23], for example, the volumetric cells type of DSC was used and the procedure was performed as follows:

The chosen compounds are dissolved in aqueous or organic solvents (usually, buffer, DMSO or 96% ethanol) and are then added to the liposomes, prepared via thin-film method as described in Section 2.1 (the final concentrations are usually in the span of 0.5–1 mg/mL of lipids). This is followed by bath-sonication of the reference and sample for 1 min, and their degassing for 20 min under a vacuum. The two DSC cells are then filled with appropriate volume of reference and sample, respectively (extra attention should be given to fill the cells without any bubbles). The correct settings are then chosen and the measurements are performed. The heating and cooling program, and the number of cycles for the measurements, are often optimised according to the aims of the study (in the case of DPPC, the samples are heated at 1 °C/min from 10–70 °C and cooled at 1 °C/min from 70–10 °C, while in the case of POPC samples are heated and cooled at 1 °C/min between −20 and 40 °C, if the machine itself allows it; altogether, two heating and 1 cooling cycles (2× heating, 1× cooling) are performed; the measurements are also performed under an additional 3 atmospheres of pressure (i.e., under 4 atmospheres)). After the measurements are completed, the cells, especially the one containing the sample, should be thoroughly cleaned, using the appropriate protocol.

In the case of organic solvents, it is critical, that the final concentrations of the solvent do not dissolve the liposomes or impact on the data in any way. This is generally achieved by maintaining solvent concentrations <1%. Furthermore, it is important to keep liposome concentrations the same throughout the experiments, to be able to fully compare the data. Anyhow, different concentrations of liposomes are used for the different types of DSC. When a volumetric cell type of DSC is used, the liposome concentrations are usually 0.5–1.0 mg/mL [18,23], whereas for pan type DSC, the concentrations between 5–10 mg/mL work best (our own observations). The solvent system used for the liposomes should be used as the reference (usually aqueous buffer), while the negative controls are the liposome suspensions without the added compounds. The first heating scan is usually used to determine the temperature of pre-transition (*T_pre_*) and gel–to–liquid phase transition (*T_m_*), as well as the model-independent calorimetric enthalpy (*ΔH_kJ/molK_*), while the subsequent heating scan is used to determine the reversibility of the lipid-phase transition. After the measurements are complete, the cells of a volumetric cell type of DSC must be fully cleaned, whereas for pan type DSC, the pans are simply discarded. Also, the samples in the pan type DSC are not exposed to additional pressure. For analysis of the data, different data processing softwares can be used, which depends on the DSC machine used (e.g., NanoAnalyze, TA Instruments, New Castle, DE, USA, for the volumetric cell type of DSC).

#### 4.5.2. Isothermal Titration Calorimetry

**Figure 5:**
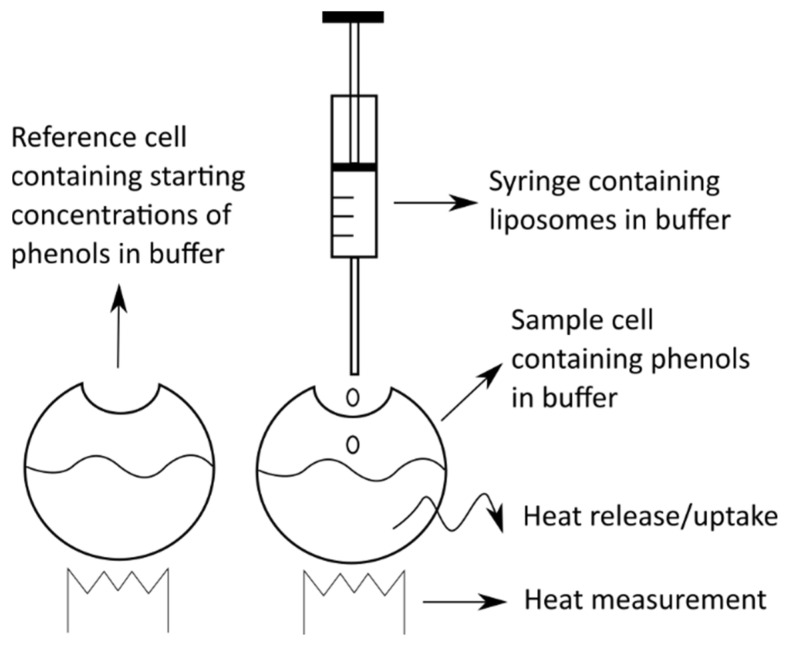
The principles and practice of isothermal titration calorimetry.

Isothermal titration calorimetry (ITC) is another calorimetric technique that can be applied for studying the interactions of different molecules with membranes. Despite being less frequently used than DSC, it still enables complex, yet different, insight into the membrane interactions, and is thus being used for such studies [94,95]. When working with ITC, the energy changes in the system derive from the changes in the concentrations of either the lipids or the compounds under investigation in the starting solutions according to a titration procedure, rather than from the changes due to temperature used, as for DSC. For example, ITC involves the gradual addition (titration) of liposomes at a certain concentration to a solution of the chosen compound (at known concentration) (Figure 5). When the liposomes and the compound meet, the compound interacts with the liposome membranes in a compound-specific way. This results in the absorption or release of energy in the form of heat. Based on this heat exchange, the amount of absorbed/released energy of the system can be calculated, and thus it is possible to define the intensity and the scale of interactions between the compound and the liposome membranes [93,96].

In the case of Witzke, Duelund [97], to study the effects of the chosen compounds (in this case terpenes) on the lipid membranes, a solution of liposomes was titrated into a solution of these compounds. The first step was to obtain liposomes in the buffer solution, which were prepared by the thin-film method and then extruded through an appropriate filter, to create LUVs or SUVs (similarly as described in Section 2.1). Their ITC measurements were obtained by injecting small aliquots of prepared liposome solution (lipid concentration, 1–20 mM) into the solution of each terpene at 0.3 mM. To interpret the data, the membrane partition coefficient (*K*) was determined, where the model used assumed that Equation (7) [97] holds for all concentrations:

(7) $$KC_{t,f}=\frac{C_{t,b}}{C_{L}}$$

where *C_L_* is the lipid concentration, and *C_t,b_* and *C_t,f_* are the concentrations of the bound and free terpene, respectively, where their sum is the total terpene injected. To determine *K*, the experimentally observed integrated heat per injection (*δh_i_*) was fitted to Equation (8) [97]:

(8) $$\delta h_{i}=C_{t}^{0}\Delta H_{t}V_{cell}\frac{K}{{\left(1+iK\delta C_{1}^{0}\right)}^{2}}+Q_{d}$$

where $C_{t}^{0}$ is the terpene concentration in the cell, Δ*H_t_* is the enthalpy for the transfer of terpene from the water into the membrane, *V_cell_* is the cell volume (1.409 mL here), *i* is the injection number, $\delta C_{1}^{0}$ is the change in lipid concentration in the cell per injection, and *Q_d_* is the heat of dilution for the injection (i.e., for both the lipid suspension and the terpene solution). Both the terpene and lipid concentrations were here corrected for dilution effects by multiplication with a dilution factor, which was calculated as (*V_cell_*)/(*V_cell_* + *iV_cell_*), where *i* is the injection number. The other thermodynamic parameters were obtained from Equations (9) and (10) [97]:

(9) $$\Delta G^{0}=-RTlnK$$

(10) ΔG=ΔH−TΔS

where Δ*G* is the change in Gibbs free energy, Δ*G*^0^ is the change in Gibbs free energy at standard conditions, *T* is the temperature in Kelvin, *K* is the partition coefficient, Δ*H* is the change in enthalpy, *R* is the universal gas constant (8.314 J/molK), and *S* is the entropy.

As such, ITC is a relatively easy method for determination of certain compound-membrane interactions, but since results acquired via DSC are easier to interpret, the latter method is used more.

## 5. Conclusions

In this article, some of the most common methods used to determine the effects of various compounds (with the emphasis on polyphenols) on the lipid membranes, as well as some of the most useful techniques for the preparation of liposomes, are reviewed. Despite the vast number of methods described here, it is still essential to note that there are also other important ones that were not mentioned (e.g., confocal microscopy), due to the sheer size of the research area. The methods described here were chosen based on their usefulness, simplicity and our experience, and could thus be described in detail and with more confidence.
